# A focus on allogeneic mesenchymal stromal cells as a versatile therapeutic tool for treating multiple sclerosis

**DOI:** 10.1186/s13287-021-02477-5

**Published:** 2021-07-13

**Authors:** Ameneh Shokati, Abdorreza Naser Moghadasi, Mohsen Nikbakht, Mohammad Ali Sahraian, Seyed Asadollah Mousavi, Jafar Ai

**Affiliations:** 1 Department of Tissue Engineering and Applied Cell Sciences, School of Advanced Technologies in Medicine, Tehran University of Medical Sciences, Tehran, Iran; 2 Multiple Sclerosis Research Center, Neuroscience Institute, Tehran University of Medical Sciences (TUMS), Tehran, Iran; 3 Hematology-Oncology and Stem Cell Transplantation Research Center, Tehran University of Medical Sciences, Tehran, Iran; 4 Hematologic Malignancies Research Center, Tehran University of Medical Sciences, Tehran, Iran

**Keywords:** Multiple sclerosis, Mesenchymal stromal cells, Stem cell therapy, Neural stem cell

## Abstract

Multiple sclerosis (MS) is a central nervous system (CNS) chronic illness with autoimmune, inflammatory, and neurodegenerative effects characterized by neurological disorder and axonal loss signs due to myelin sheath autoimmune T cell attacks. Existing drugs, including disease-modifying drugs (DMD), help decrease the intensity and frequency of MS attacks, inflammatory conditions, and CNS protection from axonal damage. As they cannot improve axonal repair and show side effects, new therapeutic options are required. In this regard, due to their neuroprotection properties, immunomodulatory effects, and the ability to differentiate into neurons, the transplantation of mesenchymal stromal cells (MSCs) can be used for MS therapy. The use of adipose-derived MSCs (AdMSCs) or autologous bone marrow MSCs (BMSCs) has demonstrated unexpected effects including the invasive and painful isolation method, inadequate amounts of bone marrow (BM) stem cells, the anti-inflammatory impact reduction of AdMSCs that are isolated from fat patients, and the cell number and differentiation potential decrease with an increase in the age of BMSCs donor. Researchers have been trying to search for alternate tissue sources for MSCs, especially fetal annexes, which could offer a novel therapeutic choice for MS therapy due to the limitation of low cell yield and invasive collection methods of autologous MSCs. The transplantation of MSCs for MS treatment is discussed in this review. Finally, it is suggested that allogeneic sources of MSCs are an appealing alternative to autologous MSCs and could hence be a potential novel solution to MS therapy.

## Introduction

MS is one of the most prevalent central nervous system (CNS)-influencing autoimmune and inflammatory neurological diseases. It induces neural fiber myelin destruction, which results in severe neurological symptoms and causes social and economic impairment in young patients, mainly between the ages of 20 and 40 [1].

The clinical signs and symptoms of MS rely on where the plaques or neural lesions are positioned and generally include tingling and numbness, imbalance of sensory, cognitive and vision impairment, fatigue, sleep and balance disorder, pains and spasms, and sexual problems [2].

Immune cell infiltration and their secretions cause inflammation in the central nervous tissues, the white and gray matter, and MS damage. Many reviews have reported that the interfering of CD4 T (T helper) cells and acquired immune reply arises from the interaction of T cells with antigen-presenting cells (APCs) that playing a primary role in the initiation and progressing of the disorder. B cells, macrophages, microglial cells (resident macrophages of the CNS), and dendritic cells (DC) are among these APCs [3].

There is still no definitive and complete treatment strategy for MS since MS’s cause is not fully understood. Treatment for MS requires a comprehensive treatment strategy. This versatility includes nutrition, rehabilitation, and medication through using of disease-modifying drugs )DMD(, including interferon-ß, fingolimod, dimethyl fumarate, and glatiramer acetate. These medications decrease the intensity and frequency of MS attacks, reduce impairment, and moderate the disease stage, while they have different side effects [4].

Stem cells (SCs) can prevent tissue degeneration as part of the natural regenerative systems of the body and regenerating the damaged tissues. They have a minimal undesirable response after injection, and most of them are safe for sick persons.

Mesenchymal stromal cells (MSCs) with self-renewal, differentiation ability, and various functional properties, such as neural differentiation potency, neurotrophic and neuroprotective ability, and anti-inflammatory impact, suggest several new tissue regeneration mechanisms used in multiple clinical trials [5].

According to the International Society of Cell Therapy (ISCT), three minimal measures for identifying hMSC include:

- MSCs are adherent to plastic if cultured in standard settings.
- Positive selection markers of MSCs include expressing CD73, CD90, and CD105. In contrast, negative selection markers of MSCs include no expression of hematopoietic markers, including B cell (CD19 or CD79a), monocyte/macrophage (CD14 or CD11b), endothelial and Pan-leukocyte marker (CD45), hematopoietic (CD34), and human leukocyte antigen -DR (HLA-DR).
- MSCs can in vitro differentiate into trilineage differentiation, chondrocytes osteoblasts, and adipocyte s[6].

Based on ISCT criteria, the isolation of MSCs has generated nonclonal, heterogeneous cultures of SCs, which contain SCs with various multipotent characteristics, committed progenitors, together with differentiated cells. The nature and functions of hMSCs are still unclear; however, sources of putative hMSCs for cell therapy are currently nonclonal stromal cultures derived from BM and alternative tissues while some reports underscore their efficacy for treating different diseases [7].

To date, many surprising data were received mostly via autologous human adipose-derived mesenchymal stromal cells (hAdMSCs) or human bone marrow-derived mesenchymal stromal cells (hBMSCs) in experimental animals and clinical trials [4, 8–10]. However, their clinical usage was restricted due to the low yield of SCs and the invasive collecting methods. As a result, these limitations have driven researchers to look for alternate sources of tissue for MSCs, including fetal annexes, which could be an innovative clinical choice for treating MS.

Here, experimental and clinical trials offer a description of MSC transplantation in MS therapy. Besides, an appealing alternative to autologous MSCs is suggested to be allogeneic sources of MSCs, which display similar qualities to autologous MSCs and are not invasive for isolation, and thus could be a potential novel solution to MS therapy [11–13].

## Mesenchymal stromal cells (MSCs)

Mesenchymal stem cells possess a broad range of diversities and consist of several sub-populations; hence it is better to call them “mesenchymal stromal cells.” These are non-hematopoietic, spindle-shaped, and self-renewable cells that are easily accessible and cultural, along with the ability to be expandable in vitro with exceptional genomic stability. They could be harvested from many tissues, including the placenta, BM, adipose, umbilical cord, peripheral blood, endometrium, Wharton’s jelly, menstrual blood, muscle, decidual, and other tissues in vitro [14].

Inflammatory cytokines, including chemokines, are elevated in damaging tissue. For example, chemokine upregulation such as CCL19, SDF-1 (stem cell-derived factor 1), CCL21, and CXCL10 stimulate the expression of their receptors, including CXCR4, CXCR3, CXCR6, CCR, and CCR10 on MSCs. The rise of MSCs receptors induces movement in the gradient of chemotactic cytokines [15].

Besides, MSCs, as the body’s natural pharmacies, have various regenerative effects due to their various microenvironments and cellular niches, differentiate and secrete diverse growth factors despite their similar phenotype and morphology [16].

**Fig. 1:**
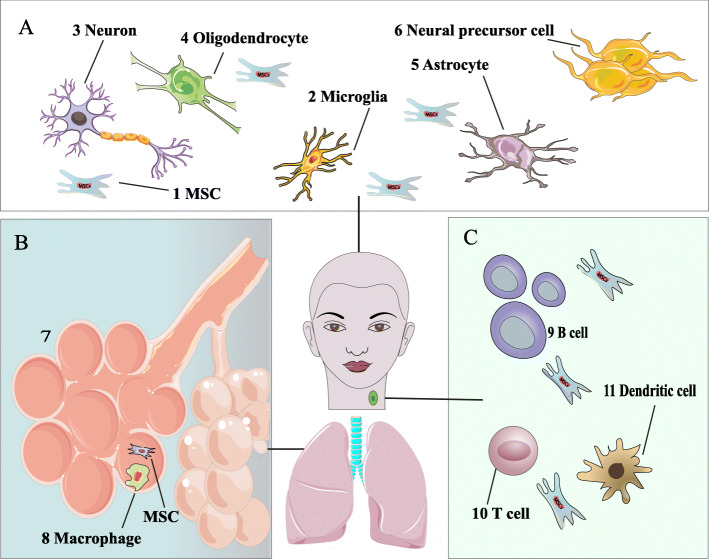
Anticipated mechanisms of action of hMSCs in neurological diseases

**Fig. 2:**
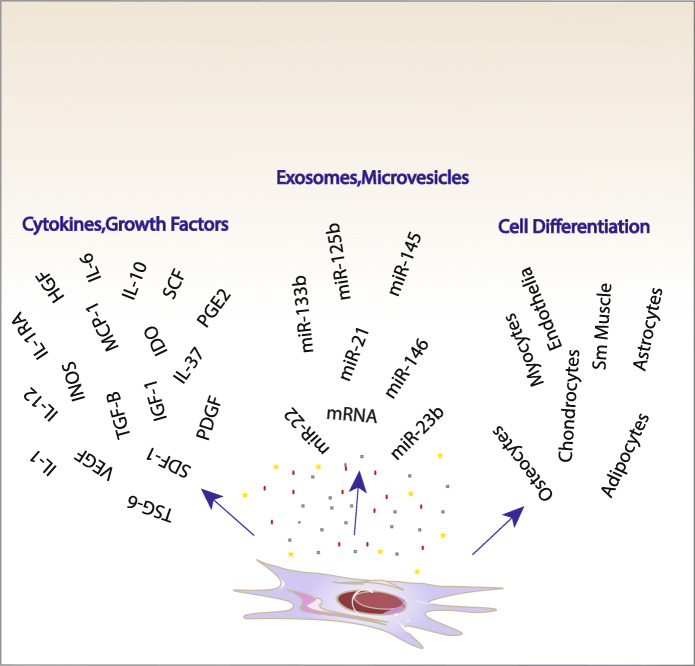
Differentiation of MSCs into osteoblasts, adipocytes, chondrocytes, and other cell lineages and their secretions such as cytokines, growth factors, and vesicle-bound molecules, as well as microRNAs, adapted from reference [17]. Reproduced by permission of the John Wiley and Sons

The proposed action mechanisms of MSCs in neurological diseases are demonstrated in Fig. 1, where:

(A) Intravenously injected MSCs could arrive at the CNS where they decline microglia proliferation after extravasation, protect neurons from degeneration which happens following oxidative, ischemic, and inflammatory damages cause remyelination by recruitment of oligodendrocytes. Moreover, they prevent the proliferation of astrocytes implicated in gliotic scarring. Interaction of resident neural precursors and MSCs could develop endogenous neurogenesis.
(B) After intravenous infusion, most MSCs are stuck in the lungs, where they are stimulated to secrete regulatory cytokines implicated in suppressing inflammation, likely by communication with local cells, including resident macrophages.
(C) MSCs could migrate to the lymph nodes, where they have various connections with immune cells: preventing the proliferation and maturation of B lymphocytes, suppressing the proliferation of T lymphocytes, inhibiting the maturation of DCs, and subsequently presenting T cells with antigens. The MSCs can differentiate into trilineage differentiation (osteoblasts, adipocytes, and chondrocytes) and other cell lineages in vitro conditions. Moreover, they can secret large amounts of vesicle-bound molecules (cytokines and growth factors), as well as microRNAs, that can signal to other tissues and cells (Fig. 2) [18].

### The impact of MSCs on B lymphocytes

B cells function as APCs in MS relapsing, introducing antigens to T cells. B cells also boost CNS responses by lymphoid follicles and secreted factors in the progressive trend of MS [19]. B-cell proliferation is repressed by MSCs, not by inducing apoptosis, but by stopping the B cell-division cycle (in the G0/G1 phase) [20].

MSCs prevent the differentiation of B cell into plasma cell and the development of antibodies; also, they inhibit B cell differentiation with a decrease in immunoglobulin (IgM, IgG, and IgA) production. Therefore, an effective mechanism of MSCs is the production and secretion of soluble factors as a paracrine leader for B cell suppression [21].

Corcione et al. [20] showed that the expression of chemokine receptors, including CXCR4, CXCR5, and CCR7, on B cell surface was downregulated by co-culture with MSCs, and decreasing them was paralleled by a reduction of B cell chemotaxis in reply to CXCL13, CXCL12, and CCL19 and the ligands of CXCR5, CXCR4, and CCR7, respectivel y[22]. Furthermore, MSC interfered with B cell function in several ways, such as chemotaxis, differentiation to antibody-producing plasma cells, and proliferation. According to the successful use and efficiency of anti-CD20 drugs that significantly target B cells, therapies that target B cells, such as MSCs injection in MS patients, can be promising [22, 23].

### The impact of MSCs on T cells

Recently, another function of MSCs has been shown that can moderate the function of many activated T cells. Earlier investigations show that MSCs inhibit T cells in a contact-dependent way with soluble factors and their two cell adhesion proteins ( VCAM-1 and ICAM-1) [21]. MSCs could hinder T CD8 and CD4 proliferation and inhibit T lymphocytes derived from other species [15]. Some influences of MSCs on T cells include:

- To delay T cell proliferation at the damage location, MSCs release hepatocyte growth factor (HGF), galectin 1, semaphoring 3A, and prostaglandin E2 (PGE2) [24].
- MSCs stimulate differentiation of T cells into CD4+ CD25+ Treg in the vicinity of immature DCs [25].
- MSCs have a dual effect with indolamine-2,3-dioxygenase (IDO) and that produces in an inflammatory condition with the presence of interferon-gamma (IFN-γ) on their surface, such as antiproliferative impacts on T cells and persuading impacts on Treg cells proliferation [26].
- MSCs modify immune responses and keeping homeostasis by releasing many cytokines by own or stimulating secreting immune cytokines [27].
- MSCs constrain naive T cell differentiation into Th17 cells. The recently identified CD4 + T h 17 subsets create IL-17 and have been involved in several autoimmunity types as an essential member of progressing disease [28].
- MSCs suppress activated T cell proliferation (TCD4 + and CD8 +) and simultaneous promotion of Treg replies as measured by increased IL10 secretion, an anti-inflammatory protein, and enhancement in Foxp3-expressing T cells (CD4 +, CD25 +) [29].

### The effect of MSCs on APCs

Previous studies have demonstrated that hMSCs affect APC, especially DCs, as potent APCs, by reducing HLA-DR expression and costimulatory molecules (CD80, CD86) [30]. Surprisingly, MSCs delay the maturation of DCs by reducing IL-12 secretion and increasing Tumor necrosis factor-inducible gene 6 protein (TSG-6), IL-10, and IL-6 production by inhibiting the activation of nuclear factor-kappa B (NF-κB) cell signaling cascade and mitogen-activated protein kinase (MAP kinase) [31].

NSCs and MSCs can express toll-like receptors (TLRs) and stimulate indoleamine 2, 3-dioxygenase 1 (IDO1) production in the immune cells. IDO1 is an immunosuppressive molecule that regulates the proliferation, differentiation, and migration of activated cells, while human MSCs stimulate through TLR ligands; further, they secret CXCL10, IL-8, and IL-6 molecule s[22]. Macrophages as DCs show similarly affected by MSCs and a sudden change to M2 activated macrophages with secreting an anti-inflammatory cytokine and decrease APC activity being showed.

**Fig. 3:**
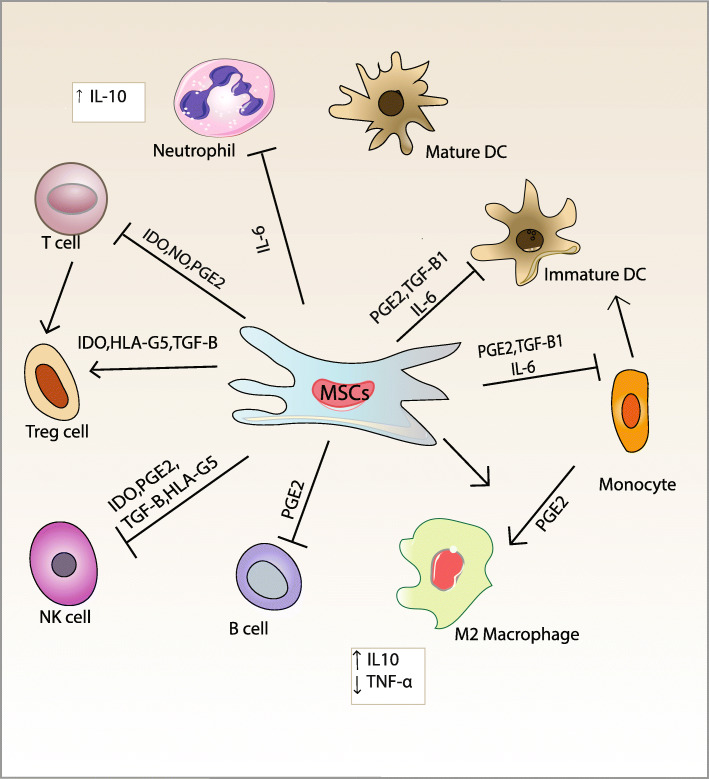
Immunomodulatory and immunosuppressive role of MSCs in the responses of immune cells, adapted from reference [33]. Reproduced by permission of Elsevier

Beyth et al. [32] exhibit that while hMSCs can raise superantigen-induced purified T lymphocytes activation, the addition of antigen-presenting cells, including APCs (either dendritic or monocytes cells) to the cultures, prevents the responses of T lymphocytes. MSCs exert an immunomodulatory and immunosuppressive role in the responses of immune cells by secreting soluble paracrine factors, as shown in Fig. 3.

### The effect of MSCs on neurons

MSCs have mesodermal lineage, but interestingly, multiple investigations have revealed to transdifferentiate these cells into ectodermal and endodermal lineages, particularly neurons and glial-like cells [34]. MSCs can also induce glial cells to produce neurotrophins, including nerve growth factor (NGF), brain-derived neurotrophic factor (BDNF), and vascular endothelial growth factor (VEGF) that could act for some of the neurodegenerative actions by influencing precursors and astrocytes through nuclear factor erythroid 2-related factor 2 (Nrf2) or p38MAPK [35].

MSCs transplantation has been demonstrated to enhance neurological functional recovery in MS preclinical animal models, including experimental autoimmune encephalomyelitis (EAE) mice [36] and non-immune CNS including stroke models [37].

**Fig. 4:**
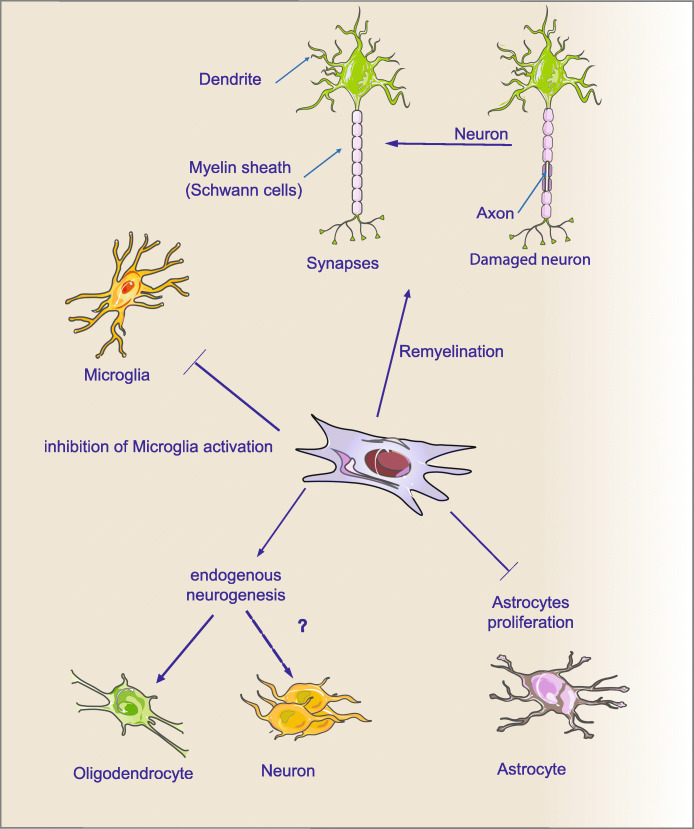
The neuroprotective and neurorestorative roles of MSCs. The dashed line indicates the lack of enough evidence for that phenomenon

Kim et al. [38] used lipopolysaccharide-induced inflammation models in vitro and in vivo to examine the protective influence of MSCs on the dopaminergic system by anti-inflammatory effects. Human MSCs therapy notably declined nitric oxide (NO) and tumor necrosis factor-alpha (TNF-α) production, inducible NO synthase mRNA expression, TNF-α, and lipopolysaccharide-induced microglial activation than the treatment group of lipopolysaccharide-only. In mesencephalic dopaminergic neurons and microglia co-cultures, hMSCs injection significantly decreased the loss of tyrosine hydroxylase-immunopositive (TH-ip) cells [39]. In Fig. 4, the neuroprotective and neurorestorative roles of MSCs are depicted. MSCs release various neurotrophic factors that induce endogenous neurogenesis, promote axonal remyelination, decrease apoptosis, and inhibit microglial activation and astrocyte proliferation.

### The effect of MSCs on inflammation

Inflammation of the CNS is one of the main pathogenic pathways in MS. Inflammation and hypoxia are vital signs of damaged tissue that can activate paracrine patterns of MSCs, which are mostly mediated via VEGF, insulin-like growth factor 1 (IGF-1), HGF, and fibroblast growth factor 2 (FGF2). Hence, when a tissue is damaged, inflammatory chemokines, including SDF-1 (CXCL12), CXCL10, CCL19, and CCL21, are increased in that tissue [40]. Increased chemokines stimulate the expression of their receptors, including CXCR4, CXCR3, CXCR10, CCR, and CCR6 on MSC, and this increase of cell surface receptors is capable of promoting immigration on the side of the chemokines gradient [41].

Therefore, MSCs with pro-inflammatory molecules (IFN-γ, IL-1β, IL-2, and TNF- α) inhibition and anti-inflammatory cytokine secretion, including IL-6, IL-10, and LIF or leukemia inhibitory factor, hinder lymphocyte activity, apoptosis, growth, and differentiation, and inflammation propagation. Meanwhile, MSCs downregulate the NF-κB signaling cascade by secreting IL-1ra and then TSG6 and reducing inflammatory cytokines’ yield [25]. In addition, releasing PGE2 by MSCs is accomplished by producing potent anti-inflammatory cytokines, like IL-10 [42].

## Autologous MSCs administration in patients with MS

### Bone marrow-MSCs (BMSCs)

Currently, BMSCs are the most regularly utilized MSCs in clinical trials. However, BMSCs have special restrictions, such as the invasive and painful isolating method used for collecting, inadequate amounts of SCs in the adult bone marrow (BM); approximately 0.001–0.01% of SC population overall, and decrease of SCs differentiation behavior and numbers with increasing the donor’s age [43].

Before they get into cellular aging and stop proliferation, BMSCs could be passaged in vitro a restricted number of times. These cells are not dead and could be retained for months in this non-proliferative stage [44]. The senescence state touches molecular and functional patterns of BMSCs are touched. This state was primarily explained by Leonard Hayflick in the 1960s [45]. Senescence molecular pathways, such as DNA damage, are triggered by increasing oxidative stress and cyclin-dependent kinase inhibitor p16INK4a, reducing the telomeres or modified telomeric structures epigenetic modifications [46].

Many intrathecal injection studies have been used BMSCs, which have shown relatively beneficial effects in patients with advanced MS [47]. In an investigation by Bonab et al. on 22 patients with advanced MS, intrathecal injection of BMSCs showed no side effects after 1 year of follow-up, while a considerable improvement or stabilization in the progressive MS disorder course was observed in patients after the first year of MSCstransplantation [48].

### Adipose-derived MSCs (AdMSCs)

Human adipose tissue is a plentiful and available source of isolating MSCs, especially for autologous SCs therapy in enough numbers with high yield (more than BM) and a minimally invasive lipectomy and discomfort method. The immunomodulatory, differentiation behaviors, and secretion profile of AdMSCs, including granulocyte-macrophage colony-stimulating factor (GM-CSF), TNF-α, IL-8, and IL-6, are equal to BMSCs. In contrast, AdMSCs are independent of age and have identical genetic stability, safety profile, viability and differentiation ability, migration, and lodging patterns in young and elderly patients in contrast to BMSCs, and are beneficial for immune-related diseases, including graft versus host disorder (GvHD), MS, and rheumatic disease [49].

Recent studies suggest that murine AdMSCs inhibited T-cells expansion via cyclooxygenase (COX-2) and inducible nitric oxide synthase (iNOS) molecules and also suppressed lipopolysaccharide (LPS)-induced DC maturation [10]. Andez et al. [50] performed a phase I/II, triple blinded, placebo-controlled, and randomized experiment with low-dose (1 × 10^6^ cells/kg) or high-dose (4 × 10^6^ cells/kg) autologous AdMSCs. They tracked the work for 12 months, showing that AdMSCs are harmless and viable in secondary progressive MS (SPMS) patients. Therefore, AdMSCs inhibit inflammation of the nervous system (NS) and increase functional improvement from traumatic brain hurt through neural stem cells.

## Allogeneic mesenchymal stromal cell transplantation in MS

Some significant benefits of using allogeneic stem cells in MS treatment include eliminating the need for patient tissue for cell isolation, along with the time needs for cell proliferation. Therefore, after autologous MSCs isolation, the appropriate number of cells is not created for the injection to patients [51].

Some patients’ cells, such as the older people, do not answer well to in vitro expansion methods, resulting in insufficient patient injection numbers. Mazzanti et al. [52] research showed the differences in MSCs cytokine profiles between healthy donors and MS patients, especially for IP10 chemokine, bound to CXCR3 reported attracting monocytes, T cells, and NK cells. Therefore, although MSCs isolated from both cell types represent the same proliferation, differentiation, toll-like receptor (TLR) marker, immunosuppressive behavior, phenotype, prevention of DC differentiation, and activation, the use of allogenic MSCs for autoimmune disorders can be more beneficial than autologous [53].

Allogeneic MSCs can be derived from perinatal origins, including umbilical cord, amniotic fluid or membrane, chorionic membrane, Wharton’s jelly, and placental decidua. Investigations showed significant advantages of the mentioned sources compared to adult sources, such as BMSCs [54].

A variety of clinical phase trials using hMSCs to treat MS have already been reported which are presented in Table 1.

**Table 1:** List of suggested or existing MSC transplantation research for MS therapy

NO	Brief title of the trial	Location	Clinical trial number	Main outcome
1.	Evaluation of autologous MSCs transplantation in MS	Royan Institute, Iran	NCT01377870	• To evaluate MRI metrics changes, brain atrophy, number of severe relapses, EDSS, MSFC, quality of life, and RAO test
2.	MSCs for MS with autologous MSCs	University of Genova, Italy	NCT01854957	• Safety • Efficacy
3.	Autologous MSCs for the treatment of MS	Karolinska University Hospital, Sweden	NCT01730547	• To measure the efficacy and safety of IV treatment with autologous MSCs as an MS therapy • Initial data collection on the effectiveness of experimental therapy in terms of combined MRI operation and therapeutic application (incidence of relapses and worsening of disabilities)
4.	BM autologous MSCs for progressive MS Sweden	Valladolid, Spain	NCT04361942	• To assess a group of patients with a harmful incidence associated with the therapy • To assess the impact of a combined number of MRI T2 lesions on the activity of MS illness • To study the impact assessed by the changes in EDSS on the activity of MS disorder • To measure the impact on populations of peripheral blood immune cells
5.	Safety and efficacy study of autologous BMSCs in MS	Amman, Jordan	NCT01895439	• To assess the number of patients with any related side effects detected • To assess the therapeutic benefits of the injected Autologous MSCs by MRI and ophthalmological tests, in addition to its safety
6.	Multi-center study safety of AdMSCs for the treatment of MS	George Town, Cayman Islands	NCT02326935	• To assess standard complication questionnaire, MS impact scale MSIS-29, improved SF-36 cell-based therapy track, physical evaluation
7.	MSCs (MESEMS) for MS	Guangzho, Guangdon, China	NCT02403947	• To determine the safety of the injection of MSCs, frequency, the timeline of incidence, and seriousness of harmful effects • To assess efficacy
8.	Feasibility study of hUC-MSCs in patients diagnosed with MS	Panama City, Panama	NCT02034188	• To evaluate some contributors with an alteration in EDSS, neurological disability as assessed by Scripps neurological rating scale, cognitive and leg function, quality of life, and alteration in MRI for spinal cord and brain
9.	MSCs transplantation in MS (CMM-EM)	Barcelona, Spain	NCT01228266	• To assess the safety and efficacy in terms of the combined amount of gadolinium-improving lesions in MRI
10.	MSCs in MS (MSCIMS)	London, UK	NCT00395200	• To assess visual ability (color and acuity), optic nerve magnetization transfer ratio, visual evoked potential latency, • To assess retinal nerve fiber layer thickness
11.	hUCMSCs transplantation for patients with Progressive MS and NMO	Nanjing, Jiangsu, China	NCT01364246	• To assess safety and efficacy • To assess adverse events
12.	autologous BMSCs injection for RRMS	Barcelona, Spain	NCT02035514	• To assess change from baseline in safety and effectiveness by MRI
13.	SCs in Rapidly Evolving Active MS (STREAMS)	London, UK	NCT01606215	• To assess the rate of recurrence, duration, and seriousness of undesirable incidence in MSC and placebo groups as determined by CTCAE v4.0 and the overall number of GELs at weeks 4, 12, and 24 after MSC treatment
14.	MSCs from autologous BM administered intravenously in patients with MS	Reina Sofia Hospital Córdoba, Spain	NCT01745783	• To evaluate the effectiveness and safety of BMSCs transplantation in participants with MS. • To evaluate the lack of unanticipated severe harmful reactions as an assessment of safety and number and size of the lesions on MRI
15.	Allogenic MSCs and physical treatment for MS	Amman, Jordan	NCT03326505	• To assess isolation and expansion of MSCs, • To evaluate safety and efficacy assessment pre- and post-treatment
16.	Autologous MSCs-derived Neural Progenitors (MSC-NP) administration in patients diagnosed with MS	New York, USA	NCT01933802	• To assess the safety, the number of participants with adverse events, and tolerability of intrathecal administration with autologous MSCNPs in MS, preliminary evaluation of efficacy
17.	Intravenous autologous MSCs administration for MS: a proof of concept Study	Ontario, Canada	NCT02239393	• To assess safety and efficacy
18.	Intrathecal administration of autologous MSC-NP in MS	New York, USA	NCT03355365	• To evaluate (EDSS) Plus MS functional composite (MSFC) and Bladder function
19.	Autologous MSC transplantation in MS	Ohio, USA	NCT00813969	• To assess the feasibility, safety, and tolerance of autologous MSC injection in participants with relapsing forms of MS
20.	Autologous BMSC-derived neural progenitors (MSC-NP) injection in progressive MS	New York, USA	NCT03822858	• To assess the tolerance and safety of autologous MSC-NP therapy with progressive MS, N:20
21.	A study of allogeneic hUC-MSC and liberation treatment in RRMS	Trinidad and Tobago	NCT02587715	• To assess the percentage of patients with medical enhancement in EDSS score in comparison with standard
22.	A study of allogeneic hUC-MSC and liberation treatment in patients with RRMS	Genesis Limited	NCT02418325	• To assess the safety, effectiveness, and adverse events
23.	Autologous AdMSCs in participants with SPMS	Sevilla, Spain	NCT01056471	• To assess tolerance and safety associated with intravenous injection of autologous MSCs
24.	Clinical efficacy of autologous BMSCs in Active & Progressive MS	Israel	NCT02166021	• To assess safety assessment, neurological efficacy
25.	NurOwn (hMSC-NTF cells) in progressive MS patients	Ohio, United States	NCT03799718	• Efficacy and safety of repeated injection (3 intrathecal dosages) of NurOwn® (MSC-NTF cells)
26.	MSCs for the treatment of MS	Guangdong, China	NCT00781872	• To assess migration capability and safety of the injected cells and clinical efficacy in disability score
27.	Autologous MSCs transplantation for MS	Barcelona, Spain	NCT02495766	• To assess adverse events, the cumulative number of MRI Gd-enhancing lesions, MS outbreaks
28.	Autologous BMSCs for the treatment of MS	Amman, Jordan	NCT03069170	• To assess efficacy by MRI and safety by vital signs, electrocardiograph monitoring, EDSS, physical inspection, analytical results, and change in quality of life
29.	SCM-010 transplantation in patients with SPMS	Tel Aviv, Israel	NCT03696485	• To assess the effectiveness and safety of rising dosages of intrathecal SCM-010 in focusses with SPMS
30	Study of autologous MSCs as a regenerative therapy for MS	University hospital of North Norway	NCT04749667	• To assess neurophysiological parameters - combined evoked potentials
31.	MSCs for progressive MS (PMS)	Karolinska Institute, Sweden	NCT03778333	• To assess the safety and adverse events of IV infusion of autologous BMSCs in PMS
32.	The effects of multiple injections of MSCs for PMS	Israel	NCT04823000	• To evaluate adverse events and EDSS
33.	The effect of autologous AdMSCs injection in the patients with SPMS	University of Mashhad, Iran	IRCT20091127002778N1	• Safety and side effects of high dose MSCs transplantation in terms of pain, hypersensitivity • Safety of SCs transplantation in terms of infection
34	BMSCs transplantation in patients with RRMS under fingolimod therapy	Tehran, Iran	IRCT20191004044975N1	• Evaluation of safety and efficacy of Intravenous and Intrathecal injection of autologous BMSCs

### Human Wharton’s jelly MSCs (WJMSCs)

The umbilical cord matrix or Wharton’s jelly is a type of tissue surrounding the umbilical blood vessels. Some investigations show that MSCs obtained from other tissues could offer theoretical benefits over BMSCs. WJMSCs show primitive nature, multi-lineage potency, immunomodulatory ability, minor immunogenic behavior, secretion of neurotrophic factors and anti-inflammatory molecules, facility of isolation, high reproduction, and without ethical concerns [55].

In the EAE model, injection of hWJMSCs-derived oligodendrocyte progenitor cells into the brain decreased the disease’s symptoms and significantly increased remyelination [13]. Donders et al. [56] showed that hWJMSC inhibited activated T cells proliferation with paracrine factors such as IDO1 and contact-dependent manner both in the EAE model rats and in vitro. Therefore, hWJMSChas trophic support behaviors and effectively moderate immune system cell functioning.

Gao et al. [57] study’s results demonstrated that WJMSCs have significant expression of undifferentiated human embryonic stem cells (hESCs) core markers, such as SOX2, LIN28, NANOG, SSEA1, SSEA4, SSEA3, KLF4, c-MYC, CRIPTO, and REX1, with a relatively lower expression than in hESCs.

Mikaeili Agah et al. [58] injected the jelly stem cell-derived oligodendrocyte progenitor cells (OPCs) of human Wharton’s jelly into the brain ventricle of an EAE-induced mice MS model. They investigated the impact of transplanted OPCs on the disease’s pathological and functional indicators. Transplanted hWJMSC-derived OPCs notably diminished clinical EAE symptoms and CNS injury of the EAE mouse model. Histological tests exhibited that remyelination was remarkably enhanced after hWJMSCs injection. Their findings indicate that hWJMSC-derived OPCs facilitate regeneration of the myelin sheath in the brain.

### Human placental MSCs (PLMSCs)

The placenta’s fetal side is three parts, including the amnion, chorion, and umbilical cord, and the maternal side includes the decidua basalis of the uterus. Researches have represented that perinatal resources of MSCs, including chorionic plate (CP), amniotic membrane (AM), decidua, and umbilical cord (UC), have benefits over adult MSCs.

It seems that decidua-derived mesenchymal stem cells (DD-MSCs) have a significant role as immune modulators in the placenta, capable of modifying lymphocyte behavior and preventing immune reactions [59].

In recent studies, PLMSCs have shown self-renewal capacity and multi-lineage differentiation, no ethical problems, ease accessibly, abundant, and strongly powerfully immunosuppressive behavior isolated from discarding pregnancy products without invasive procedures. In comparison, the extra-embryonic placental tissue is an excellent mass and is readily processed relative to the BM or adipose tissues and creates high quantities of MSCs [60].

Clark et al*.* reported that PLMSCs-derived extracellular vesicles improve myelin regeneration in EAE models. The injection of PLMSCs significantly decreased the mean clinical score, inflammatory process, and neural demyelination in EAE mice of MS. Moreover, the concentration of IL-23, as a pro-inflammatory cytokine, was reduced. Additionally, the concentration of IL-27, as an anti-inflammatory cytokine, was enhanced in the serum. IL-23 and IL-27 cytokines are associated with excellent and poor MS disease prognosis, respectively [61].

The placental MSC-derived extracellular vesicles that facilitate myelin regeneration were investigated by Clark et al. in an animal MS model. Their in vitro data showed that by promoting OPCs to change into myelinating mature oligodendroglia or oligodendrocyte cells, placental MSC-derived extracellular vesicles facilitate myelin regeneration. These results confirm that the secretion of EVs mediates the action process of PMSCs. So, EVs derived from PMSC are a practical option for multiple sclerosis cellular-based therapies [62].

### Human umbilical cord MSCs (hUC-MSCs)

Human umbilical cord MSCs are heterogeneous cell populations, such as 1 and 2 type in culture, and have differential filaments (vimentin and cytokeratin). They are potential cells for cell therapy because of their collection without pain or invasive method, higher and faster proliferation, differentiation abilities, and more vital immune tolerance due to lower HLA-1 expression than other MSCs, especially BMSCs. Also, they differentiate into vast cell types, such as trilineage differentiation, muscle, cardiomyocyte, astrocyte, neuron, and oligodendrocyte.

BMSCs can easily differentiate from adipocyte cells, whereas they have less potency in forming chondrogenic and osteogenic cells than hUC-MSC. Moreover, hUC-MSCs can express immature neuronal markers [63].

Furthermore, an enhancement of HGF factor was seen in the UC-MSCs-treated group, which can have played an important role in improving MS disease. It is necessary to mention that HGF is a multi-functional mediator for the regeneration of tissue with its ability to induce matrix invasion, cell motility, and mitogenesis. In vivo and in vitro experiments have shown that hUC-MSCs have not been transformed into tumor-associated fibroblasts, rendering them safer than BMSCs [64].

## Challenges in MSC-based therapy

The most vital dangers of MSCs are fibrosis, proinflammation, and tumorigenicity which is considered the leading risk factor of MSCs in clinical applications. MSCs can migrate to cancer environments through several chemokines, including CXCR4, CXCL7, CXCL6, and CXCL5, along with inflammatory molecules, such as TNF-α, IFN-γ, and IL-6 and growth factors like platelet-derived growth factor (PDGF) and HGF [65].

MSCs have been linked to dual effects of progression and suppression on tumor growth, which is also observed in the angiogenic process. Neo-angiogenesis is possible probably due to settling into the walls of tumor vessels after engraftment, enhancing tumor proliferation due to the pro-angiogenic profiles stimulation, and differentiate into pericytes and endothelial cells in vessels. Additionally, MSCs probably stimulate hypoxia-related gene expression and epithelial-mesenchymal transition (EMT) in tumor cells and elevate cancer cell invasion.

In contrast, in other tumor-based studies, MSCs have been indicated to suppress cancer progress by activating programmed cell death in endothelial cells [66].

Furthermore, the therapeutic promise of MSCs in inflammatory and injured tissues probably rely on numerous features, such as the stem cells’ quantity, the passage number of transplanted cells, the injection time and method, cell engraftment frequency, cryopreserved duration of MSCs, type, stage, the disease’s involved cells, the protocol for in vitro culture, such as using of fetal bovine serum (FBS) or essential supplements, the proper cells’ dose, induction factors, oxygen concentrations, and mechanical stimuli [67].

## Conclusions and future insights

The potency of MSCs, such as MS, has attracted significant attention for systemic transplantation of these cells to treat CNS disorders. Although most proposals persist on autologous MSCs injection for use in autoimmune disorders, and especially MS, this study concluded that, due to relative safety, uncomplicated manufacturing procedure, the ability to isolate readily, low immunogenicity, independent to the donors’ age, less ethical considerations, secretion of relevant factors, less invasive than autologous stem cells, and easy expansion and in vitro manipulation to reach the pureness and essential amounts for transplantation, allogeneic transplantation could be considered a superior option for MS treatment.

## Data Availability

Not applicable.

## Abbreviations

AD-MSCs: Adipose-derived mesenchymal stem cells
APC: Antigen-presenting cells
BBB: Blood-brain barrier
BDNF: Brain-derived neurotrophic factor
BM-MSCs: Bone marrow mesenchymal stem cells
BMP-4: Morphogenic protein-4
CCR: Chemokine (c-c motif) receptor
CNS: Central nervous system
CTLs: Cytotoxic T lymphocytes
CXCR: Chemokine (c-x-c motif) receptor
DCs: Dendritic cells
DD-MSCs: Decidua-derived mesenchymal stem cells
EAE: Experimental autoimmune encephalomyelitis
EDSS: Expanded disability status scale
ESCs: Embryonic stem cells
FBS: Fetal bovine serum
FGF2: Fibroblast growth factor 2
FOXP3: Forkhead box P3
G-CSF: Granulocyte colony stimulating factor
GDNF: Glial cell line derived growth factor
HGF: Hepatocyte growth factor
HLA: Human leukocyte antigen
HSCs: Hematopoietic stem cells
HSCT: Hematopoietic stem cell transplantation
hUC-MSCs: Human umbilical cord-derived MSCs
IDO1: Indolamine-2,3-dioxygenase 1
IFN-γ: Interferon-gamma
IL-1: Interleukin-1
INF: Interferon
KLF4: Kruppel-like factor four4
LIF: Leukemia inhibitory factor
MHC-I: Major histocompatibility complex class I
MHC-II: Major histocompatibility complex class II
MS: Multiple sclerosis
MRI: Magnetic resonance imaging
NCSCs: Neural crest stem cells
NGF: Neural growth factor
NK: Natural killer
NSC: Neural stem cells
PGE-2: Prostaglandin
PLMSCs: Placenta-derived mesenchymal stem cells
SCs: Stem cells
SDF-1: Stromal-derived factor-1
TC: Cytotoxic T
TLR: Toll-like receptor
Treg: T regulatory
VCAM-1: Vascular cellular adhesion molecule
VEGF: Vascular endothelial growth factor
VLA-4: Very late activation antigen-4
WJ-MSCs: Wharton’s jelly mesenchymal stem cells.

## References

[1] Dobson R, Giovannoni G (2019). Multiple sclerosis – a review. Eur J Neurol.

[2] Azami M, YektaKooshali MH, Shohani M, Khorshidi A, Mahmudi L (2019). Epidemiology of multiple sclerosis in Iran: A systematic review and meta-analysis. PLoS ONE.

[3] Dendrou CA, Fugger L, Friese MA (2015). Immunopathology of multiple sclerosis. Nat Rev Immunol.

[4] Sahraian MA, Mohyeddin Bonab M, Baghbanian SM, Owji M, Naser Moghadasi A (2019). Therapeutic use of intrathecal mesenchymal stem cells in patients with multiple sclerosis: a pilot study with booster injection. Immunol Investig.

[5] Astaneh ME, Goodarzi A, Khanmohammadi M, Shokati A, Mohandesnezhad S, Ataollahi MR, Najafipour S, Farahani MS, Ai J (2020). Chitosan/gelatin hydrogel and endometrial stem cells with subsequent atorvastatin injection impact in regenerating spinal cord tissue. J Drug Deliv Sci Technol.

[6] Dominici M, Le Blanc K, Mueller I, Slaper-Cortenbach I, Marini F, Krause D, Deans R, Keating A, Prockop D, Horwitz E (2006). Minimal criteria for defining multipotent mesenchymal stromal cells. The International Society for Cellular Therapy position statement, Cytotherapy.

[7] Squillaro T, Peluso G, Galderisi U (2016). Clinical trials with mesenchymal stem cells: an update. Cell Transplant.

[8] Cristofanilli M, Harris VK, Zigelbaum A, Goossens AM, Lu A, Rosenthal H, Sadiq SA (2011). Mesenchymal stem cells enhance the engraftment and myelinating ability of allogeneic oligodendrocyte progenitors in dysmyelinated mice. Stem Cells Dev.

[9] Fernández O, Izquierdo G, Fernández V, Leyva L, Reyes V, Guerrero M, León A, Arnaiz C, Navarro G, Páramo MD (2018). Adipose-derived mesenchymal stem cells (AdMSC) for the treatment of secondary-progressive multiple sclerosis: A triple blinded, placebo controlled, randomized phase I/II safety and feasibility study. PLoS One.

[10] Anderson P, Gonzalez-Rey E, O’Valle F, Martin F, Oliver FJ, Delgado M. Allogeneic adipose-derived mesenchymal stromal cells ameliorate experimental autoimmune encephalomyelitis by regulating self-reactive T cell responses and dendritic cell function. Stem Cells Int. 2017;2017. 10.1155/2017/2389753 PMC5303870 28250776

[11] Yousefi F, Ebtekar M, Soleimani M, Soudi S, Hashemi SM (2013). Comparison of in vivo immunomodulatory effects of intravenous and intraperitoneal administration of adipose-tissue mesenchymal stem cells in experimental autoimmune encephalomyelitis (EAE). Int Immunopharmacol.

[12] Shigeno Y, Ashton BA (1995). Human bone-cell proliferation in vitro decreases with human donor age. J Bone Joint Surg Br.

[13] Kim HJ, Park J-S (2017). Usage of human mesenchymal stem cells in cell-based therapy: advantages and disadvantages. Dev Reprod.

[14] Horwitz EM, Keating A (2000). Nonhematopoietic mesenchymal stem cells: What are they?. Cytotherapy.

[15] Lapidot T, Dar A, Kollet O. Review article How do stem cells find their way home? 2005;106(6):1901–10. 10.1182/blood-2005-04-1417 15890683

[16] Lin H (2002). The stem-cell niche theory: Lessons from flies. Nat Rev Genet.

[17] Phinney DG, Pittenger MF (2017). Concise review: MSC-derived exosomes for cell-free therapy. Stem Cells.

[18] Uccelli A, Laroni A, Freedman MS (2011). Mesenchymal stem cells for the treatment of multiple sclerosis and other neurological diseases. Lancet Neurol.

[19] Baecher-Allan C, Kaskow BJ, Weiner HL (2018). Multiple Sclerosis: Mechanisms and Immunotherapy. Neuron.

[20] Corcione A, Benvenuto F, Ferretti E, Giunti D, Cappiello V, Cazzanti F, Risso M, Gualandi F, Mancardi GL, Pistoia V, Uccelli A (2006). Human mesenchymal stem cells modulate B-cell functions. Blood.

[21] Darlington PJ, Boivin MN, Bar-Or A (2011). Harnessing the therapeutic potential of mesenchymal stem cells in multiple sclerosis. Expert Rev Neurother.

[22] Baker D, Marta M, Pryce G, Giovannoni G, Schmierer K (2017). Memory B cells are major targets for effective immunotherapy in relapsing multiple sclerosis. EBioMedicine.

[23] Van Langelaar J, Rijvers L, Smolders J, van Luijn MM. B and T cells driving multiple sclerosis: identity, mechanisms and potential triggers. Front Immunol. 2020;11. 10.3389/fimmu.2020.00760 PMC7225320 32457742

[24] C.T.c. Immunosuppression, Lepelletier Y, Lecourt S, Renand A, Arnulf B, Vanneaux V, et al. Galectin-1 and Semaphorin-3A Are Two Soluble Factors. Stem Cells Dev. 2010;19(7). 10.1089/scd.2009.0212 19886821

[25] Roncarolo BM-G, Levings MK. Differentiation of T Regulatory Cells by Immature Dendritic Cells. J Exp Med. 2001. 10.1084/jem.193.2.f5 PMC2193342 11208869

[26] Gebler A, Zabel O, Seliger B (2012). The immunomodulatory capacity of mesenchymal stem cells. Trends Mol Med.

[27] Selmani Z, Naji A, Zidi I, Favier B, Gaiffe E, Obert L, Borg C, Saas P, Tiberghien P, Rouas-Freiss N, Carosella ED, Deschaseaux F (2008). Human Leukocyte Antigen-G5 Secretion by Human Mesenchymal Stem Cells Is Required to Suppress T Lymphocyte and Natural Killer Function and to Induce CD4 + CD25 high FOXP3 + Regulatory T Cells. Stem Cells.

[28] Duffy MM, Pindjakova J, Hanley SA, McCarthy C, Weidhofer GA, Sweeney EM, English K, Shaw G, Murphy JM, Barry FP, Mahon BP, Belton O, Ceredig R, Griffin MD (2011). Mesenchymal stem cell inhibition of T-helper 17 cell- differentiation is triggered by cell-cell contact and mediated by prostaglandin E2 via the EP4 receptor. Eur J Immunol.

[29] Ostrand-Rosenberg S, Horn LA, Haile ST (2014). The Programmed Death-1 Immune-Suppressive Pathway: Barrier to Antitumor Immunity. J Immunol.

[30] Arneth BM (2019). Impact of B cells to the pathophysiology of multiple sclerosis. J Neuroinflammation.

[31] Alsaab HO, Sau S, Alzhrani R, Tatiparti K, Bhise K, Kashaw SK, et al. PD-1 and PD-L1 Checkpoint Signaling Inhibition for Cancer Immunotherapy: Mechanism, Combinations, and Clinical Outcome. Front Pharmacol. 2017;8. 10.3389/fphar.2017.00561 PMC5572324 28878676

[32] Beyth S, Borovsky Z, Mevorach D, Liebergall M, Gazit Z, Aslan H, Galun E, Rachmilewitz J (2005). Human mesenchymal stem cells alter antigen-presenting cell maturation and induce T-cell unresponsiveness. Blood.

[33] Regmi S, Pathak S, Kim JO, Yong CS, Jeong J-H (2019). Mesenchymal stem cell therapy for the treatment of inflammatory diseases: Challenges, opportunities, and future perspectives. Eur J Cell Biol.

[34] Bonab MM, Shakiba Y, Talebian F, Nikbin B (2015). Mechanisms and potentials of stem cells in the treatment of multiple sclerosis: The unpaved path.

[35] Uccelli A, Benvenuto F, Laroni A, Giunti D (2011). Neuroprotective features of mesenchymal stem cells. Best Pract Res Clin Haematol.

[36] Zhang J, Li Y, Chen J, Cui Y, Lu M, Elias SB, Mitchell JB, Hammill L, Vanguri P, Chopp M (2005). Human bone marrow stromal cell treatment improves neurological functional recovery in EAE mice. Exp Neurol.

[37] Wu X, Jiang J, Gu Z, Zhang J, Chen Y, Liu X (2020). Mesenchymal stromal cell therapies: Immunomodulatory properties and clinical progress. Stem Cell Res Ther.

[38] Kim YJ, Park HJ, Lee G, Bang OY, Ahn YH, Joe E, Kim HO, Lee PH (2009). Neuroprotective effects of human mesenchymal stem cells on dopaminergic neurons through anti-inflammatory action. Glia.

[39] L.D. Liangyu Lin, The role of secreted factors in stem cells-mediated immune regulation. 10.1016/j.cellimm.2017.07.010 28778535

[40] Ghaffari-Nazari H (2018). The known molecules involved in MSC homing and migration. J Stem Cell Res Med.

[41] Sordi V, Malosio ML, Marchesi F, Mercalli A, Melzi R, Giordano T, Belmonte N, Ferrari G, Leone BE, Bertuzzi F, Zerbini G, Allavena P, Bonifacio E, Piemonti L (2005). Bone marrow mesenchymal stem cells express a restricted set of functionally active chemokine receptors capable of promoting migration to pancreatic islets. Blood.

[42] Prockop DJ, Youn Oh J (2012). Mesenchymal stem/stromal cells (MSCs): Role as guardians of inflammation. Mol Ther.

[43] Cova L, Bossolasco P, Armentero MT, Diana V, Zennaro E, Mellone M, Calzarossa C, Cerri S, Lambertenghi Deliliers G, Polli E, Blandini F, Silani V (2012). Human bone-cell proliferation in vitro decreases with human donor age. Apoptosis.

[44] Ganjei J (1997). Usage of Human Mesenchymal Stem Cells in Cell-based Therapy: Advantages and Disadvantages.

[45] Duscher D, Rennert RC, Januszyk M, Anghel E, Maan ZN, Whittam AJ, et al. Aging disrupts cell subpopulation dynamics and diminishes the function of mesenchymal stem cells. Sci Rep. 2014;4(December). 10.1038/srep07144 PMC4239576 25413454

[46] Spack E (2016). Regenerative Medicine -from Protocol to Patient II.

[47] Mohyeddin Bonab M, Ali Sahraian M, Aghsaie A, Ahmadi Karvigh S, Massoud Hosseinian S, Nikbin B, Lotfi J, Khorramnia S, Reza Motamed M, Togha M, Hossien Harirchian M, Beladi Moghadam N, Alikhani K, Yadegari S, Jafarian S, Reza Gheini M (2012). Autologous Mesenchymal Stem Cell Therapy in Progressive Multiple Sclerosis: An Open Label Study. Curr Stem Cell Res Ther.

[48] Zhou C, Yang B, Tian Y, Jiao H, Zheng W, Wang J, Guan F (2011). Immunomodulatory effect of human umbilical cord Wharton ’ s jelly-derived mesenchymal stem cells on lymphocytes. Cell Immunol.

[49] Guilak F (2017). Adipose-derived adult stem cells : isolation , characterization , and differentiation potential, (January 2003).

[50] Fernández O, Izquierdo G, Fernández V, Leyva L, Reyes V, Guerrero M, León A, Arnaiz C, Navarro G, Páramo MD, De la Cuesta A, Soria B, Hmadcha A, Pozo D, Fernandez-Montesinos R, Leal M, Ochotorena I, Gálvez P, Geniz MA, Barón FJ, Mata R, Medina C, Caparrós-Escudero C, Cardesa A, Cuende N (2018). Adipose-derived mesenchymal stem cells (AdMSC) for the treatment of secondary-progressive multiple sclerosis: A triple blinded, placebo controlled, randomized phase I/II safety and feasibility study.

[51] Kim J-H, Jo CH, Kim H-R, Hwang Y-i (2018). Comparison of Immunological Characteristics of Mesenchymal Stem Cells from the Periodontal Ligament. Umbilical Cord, and Adipose Tissue, Stem Cells International.

[52] Mazzanti B, Aldinucci A, Biagioli T, Barilaro A, Urbani S, Dal Pozzo S, Amato MP, Siracusa G, Crescioli C, Manuelli C, Bosi A, Saccardi R, Massacesi L, Ballerini C (2008). Differences in mesenchymal stem cell cytokine profiles between MS patients and healthy donors: Implication for assessment of disease activity and treatment. J Neuroimmunol.

[53] Baggiolini M, Dewald B, Moser B (1997). HUMAN CHEMOKINES : An Update.

[54] Rafei M, Birman E, Forner K, Galipeau J (2009). Allogeneic mesenchymal stem cells for treatment of experimental autoimmune encephalomyelitis. Mol Ther.

[55] Frausin S, Viventi S, Verga L, Jlenia M, Leanza G, Tommasini A, et al. Wharton's jelly derived mesenchymal stromal cells: Biological properties, induction of neuronal phenotype and current applications in neurodegeneration research. Acta Histochem. 2015. 10.1016/j.acthis.2015.02.005 25747736

[56] Donders R, Vanheusden M, Bogie JFJ, Ravanidis S, Thewissen K (2015). Therapeutic effect of transplanted human Wharton’s jelly stem cell-derived oligodendrocyte progenitor cells (hWJ-MSC-derived OPCs) in an animal model of mul tiple sclerosis.

[57] Gao LR, Zhang NK, Ding QA, Chen HY, Hu X, Jiang S, Li TC, Chen Y, Wang ZG, Ye Y, Zhu ZM (2013). Common Expression of Stemness Molecular Markers and Early Cardiac Transcription Factors in Human Wharton ’ s Jelly-Derived Mesenchymal Stem Cells and Embryonic Stem Cells. Cell Transplant.

[58] Mikaeili Agah E, Parivar K, Joghataei MT (2014). Therapeutic effect of transplanted human Wharton's jelly stem cell-derived oligodendrocyte progenitor cells (hWJ-MSC-derived OPCs) in an animal model of multiple sclerosis. Mol Neurobiol.

[59] Vinketova K, Mourdjeva M, Oreshkova T. Human Decidual Stromal Cells as a Component of the Implantation Niche and a Modulator of Maternal Immunity. J Pregnancy. 2016;2016(Figure 1). 10.1155/2016/8689436 PMC4864559 27239344

[60] Kim GJ (2015). Advanced Research on Stem Cell Therapy for Hepatic Diseases: Potential Implications of a Placenta-derived Mesenchymal Stem Cell-based Strategy. Hanyang Med Rev.

[61] Jazayeri MH, Barzaman K, Nedaeinia R, Aghaie T, Motallebnezhad M (2020). Human placental extract attenuates neurological symptoms in the experimental autoimmune encephalomyelitis model of multiple sclerosis-A putative approach in MS disease?. Autoimmunity Highlights.

[62] Clark K, Zhang S, Barthe S, Kumar P, Pivetti C, Kreutzberg N, Reed C, Wang Y, Paxton Z, Farmer D, Guo F, Wang A (2019). Placental Mesenchymal Stem Cell-Derived Extracellular Vesicles Promote Myelin Regeneration in an Animal Model of Multiple Sclerosis.

[63] Cinar O, Kilic E, Uckan D, Demiralp DO (2007). Biology of Stem Cells in Human Umbilical Cord Stroma. Situ and In Vitro T ISSUE -S PECIFIC S TEM C ELLS.

[64] Subramanian A, Gan SU, Ngo KS, Gauthaman K, Biswas A, Choolani M, Bongso A, Fong CY (2012). Human umbilical cord Wharton's jelly mesenchymal stem cells do not transform to tumor-associated fibroblasts in the presence of breast and ovarian cancer cells unlike bone marrow mesenchymal stem cells. J Cell Biochem.

[65] Arango-Rodriguez ML (2015). Could cancer and infection be adverse effects of mesenchymal stromal cell therapy?. World J Stem Cells.

[66] Kéramidas M, De Fraipont F, Karageorgis A, Moisan A, Persoons V, Richard MJ, Coll JL, Rome C (2013). The dual effect of mscs on tumour growth and tumour angiogenesis. Stem Cell Res Ther.

[67] Barati S, Tahmasebi F, Faghihi F (2020). Effects of mesenchymal stem cells transplantation on multiple sclerosis patients. Neuropeptides.

